# Supplementary material for: Limitations of gene editing assessments in human preimplantation embryos
Source: Nat Commun. 2023 Mar 7;14:1219. doi: 10.1038/s41467-023-36820-6 (PMC9992379; doi:10.1038/s41467-023-36820-6)
Supplement: Supplementary file 1 — Supplementary Information [file 41467_2023_36820_MOESM1_ESM.pdf]

Supplementary Information

Limitations of gene editing assessments in human preimplantation embryos

Dan Liang[1,2] *, Aleksei Mikhalchenko[1]*, Hong Ma[1], Nuria Marti Gutierrez[1], Tailai Chen[3], Yeonmi Lee[4,5], Sang-Wook Park[6,7], Rebecca Tippner-Hedges[1], Amy Koski[1], Hayley Darby[1], Ying Li[1], Crystal Van Dyken[1], Han Zhao[3], Keliang Wu[3], Jingye Zhang[3], Zhenzhen Hou[3], Seongjun So[4], Jongsuk Han[4], Jumi Park[5], Chong-Jai Kim[5], Kai Zong[8], Jianhui Gong[9,10], Yilin Yuan[9,10], Ying Gu[9,10], Yue Shen[9,10], Susan B.Olson[11], Hui Yang[12], David Battaglia[1], Thomas O'Leary[13], Sacha A. Krieg[13], David M. Lee[13], Diana H.Wu[13], P. Barton Duell[14], Sanjiv Kaul[14], Jin-Soo Kim[6,15], Stephen B. Heitner[14], Eunju Kang[4,5], Zi-Jiang Chen[1,3,16], Paula Amato[1,13#], and Shoukhrat Mitalipov[1#]

* These authors contributed equally.

# These authors jointly supervised this work. Correspondence: shoukhrat@gmail.com

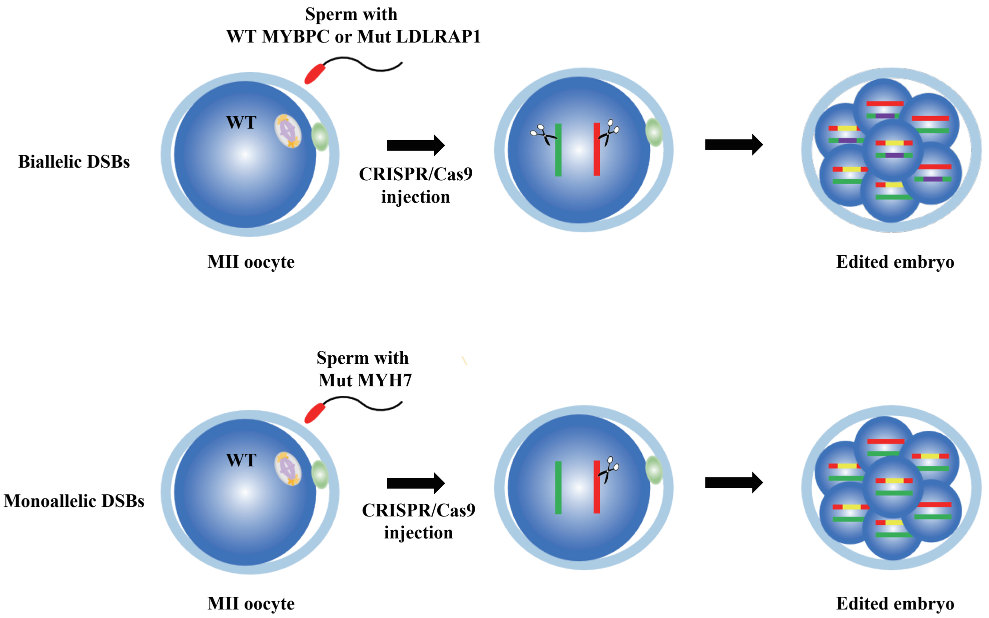

Supplementary Fig. 1: Schematic of DSB repair in human embryos. Upper: Schematic of biallelic DSBs induced by injection of CRISPR/Cas9 into human zygotes fertilized with *MYBPC3* WT or *LDLRAP1* mutant sperm. Embryos at the 4–8-cell stage were collected for genetic analysis. Injection resulted in mosaic embryos consisting of various edited sister blastomeres. Lower: Schematic of monoallelic DSBs induced by injection of CRISPR/Cas9 into heterozygous *MYH7* zygotes.

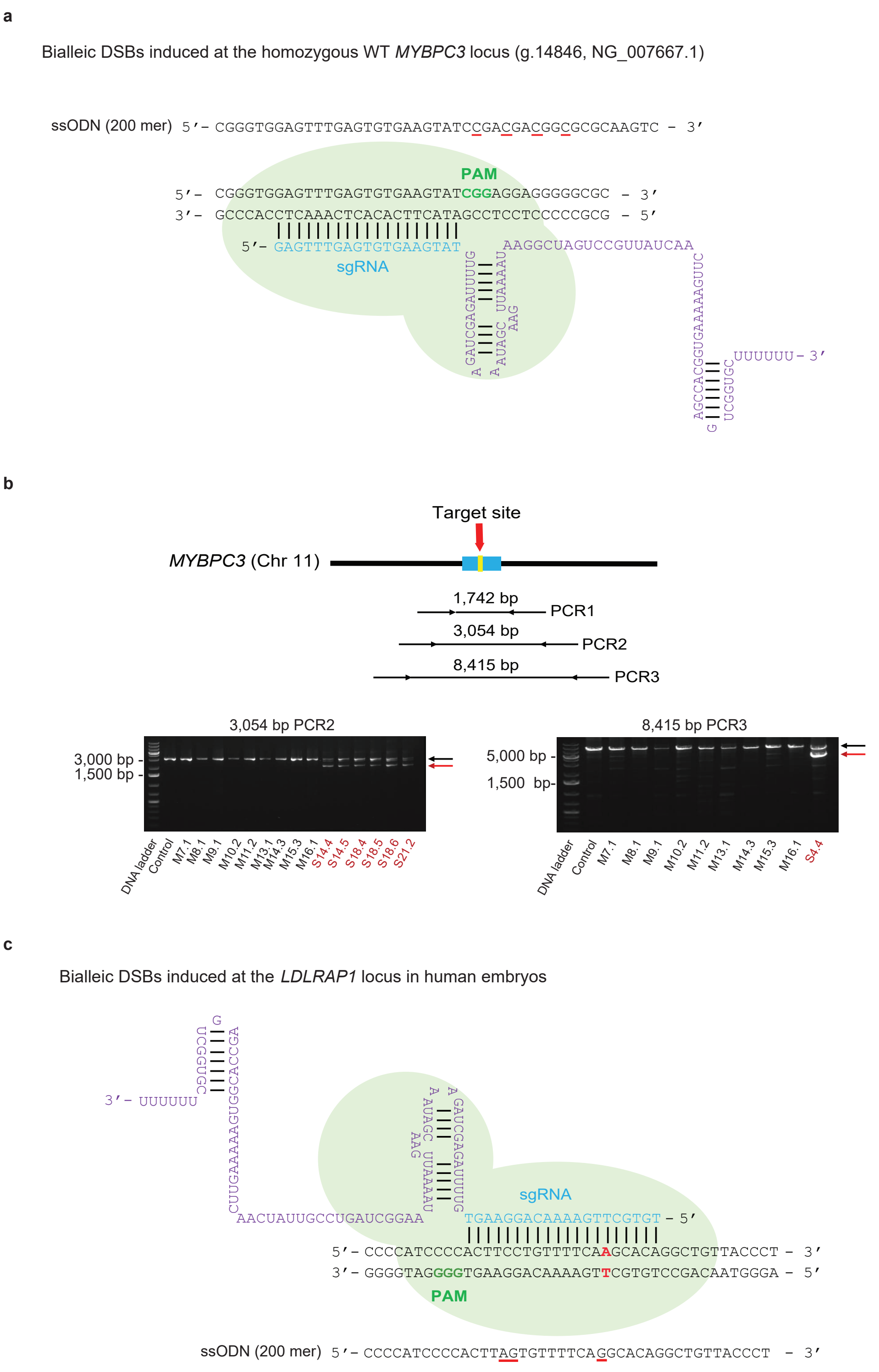

Supplementary Fig. 2: DSBs induced at *MYBPC3* and *LDLRAP1* loci and long-range PCR for detection of large deletions. **a.** Human wildtype *MYBPC3* target locus. Pre-selected sgRNA targets both wild type alleles (biallelic DSBs). Green font nucleotides indicate PAM, blue font nucleotides indicate sgRNA, red underlined nucleotides show substitutions in ssODN. **b.** Representative agarose gel of long-range PCR screening for large deletions in *MYBPC3* region. DNA from individual blastomeres (n=16 in CRISPR/Cas9 targeted blastomeres, n=1 in control blastomere) was analyzed using 3 pairs of long-range PCR primers spanning the target *MYBPC3* locus. Note that 6 blastomeres (shown in red fonts) with *MYBPC3*$^{Del/Indel}$ genotype showed a secondary band indicating large deletions (gel PCR2, pointed by red arrows). Likewise, one blastomere (S4.4, shown by red fonts in PCR3 gel) with *MYBPC3*$^{Del/Indel}$ genotype had a large deletion (pointed by red arrow). Black arrows show expected size PCR brands. See more details in **Supplementary Table 1. c.** Pre-selected sgRNA targets both WT and mutant *LDLRAP1* alleles in human embryos (biallelic DSBs). Green font nucleotides indicate PAM, blue font nucleotides indicate sgRNA, red underlined nucleotides show substitutions in ssODN. Source data are provided as Source Data Files.

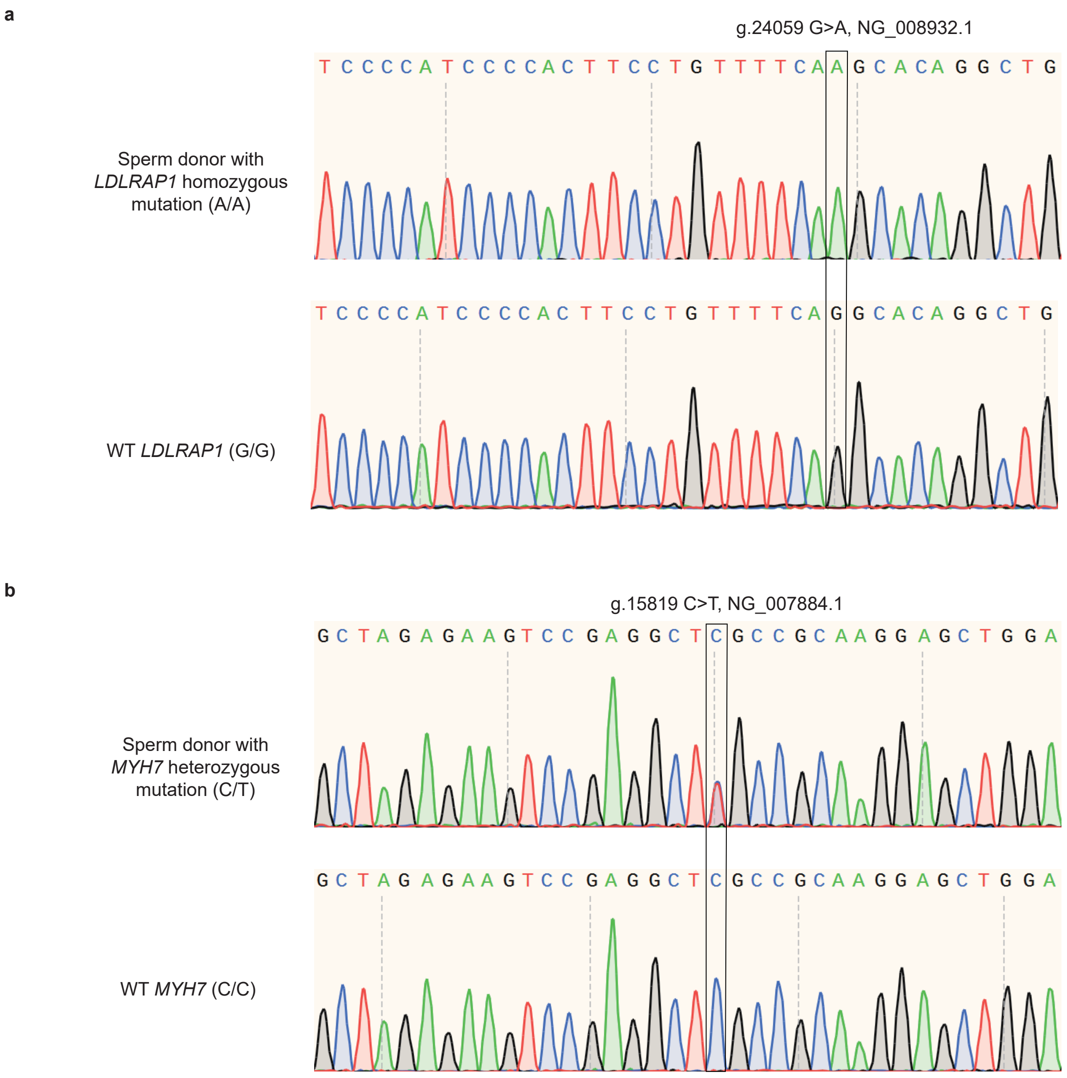

Supplementary Fig. 3: Chromatograms of Sanger sequencing for *LDLRAP1* and *MYH7* mutations. **a.** Chromatograms demonstrating homozygous *LDLRAP1 G>A* mutation locus in a sperm donor. **b.** Chromatograms demonstrating heterozygous *MYH7 C>T* mutation locus in a sperm donor.

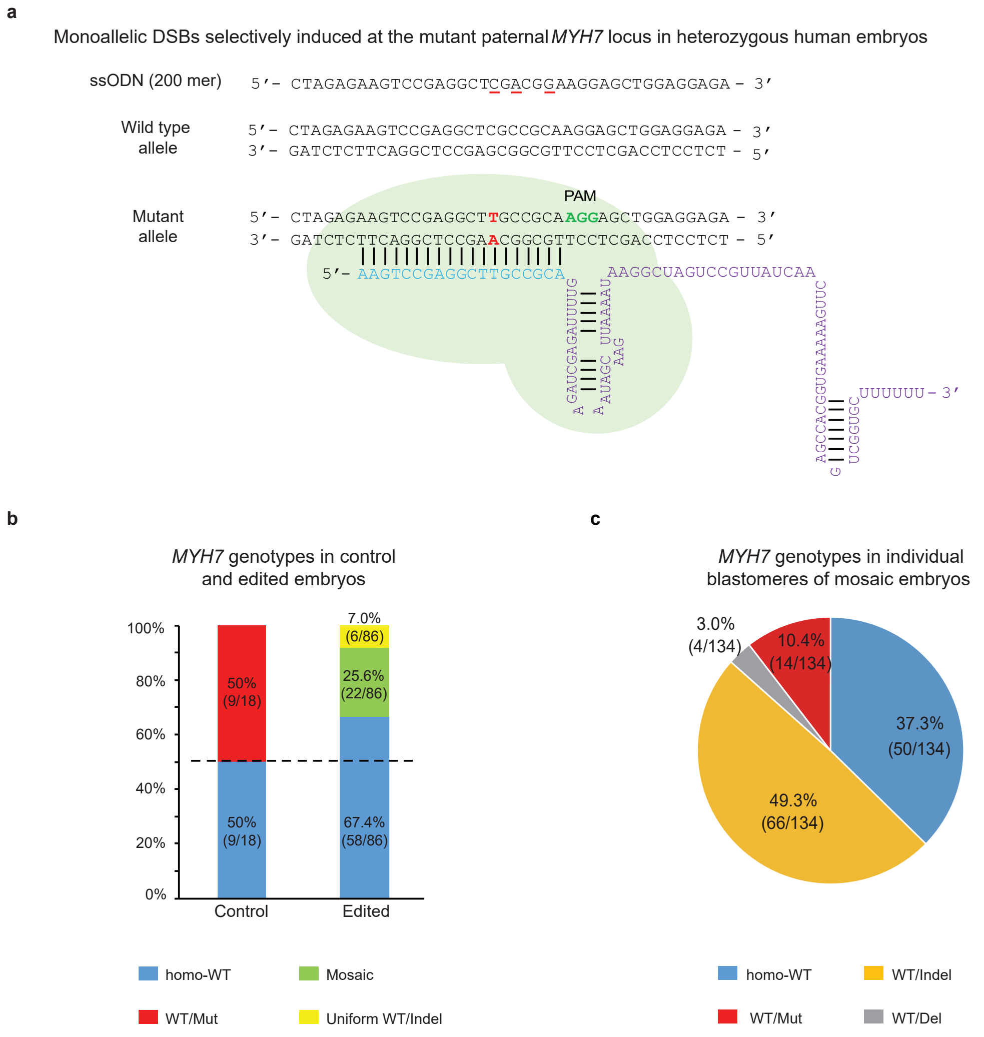

Supplementary Fig. 4: Monoallelic DSBs at *MYH7* locus and repair outcomes in human embryos. **a.** Human wildtype and mutant *MYH7 g.15819 C>T* locus implicated in hypertrophic cardiomyopathy. sgRNA was designed to target the mutant allele (monoallelic DSBs). Red font nucleotides show mutation locus, green indicate PAM, and red underlined nucleotides show substitutions in ssODN. **b.** Genotypes at the *MYH7* target region in control and edited human embryos. Half of the control embryos were *MYH7*$^{homo\text{-}WT}$ homozygous while other half were *MYH7*$^{WT/Mut}$ heterozygous, typical for sperm from the heterozygous subject. **c.** *MYH7* target region genotypes in individual blastomeres of mosaic embryos. Source Data are provided as Supplementary Data 3 and 4.

a

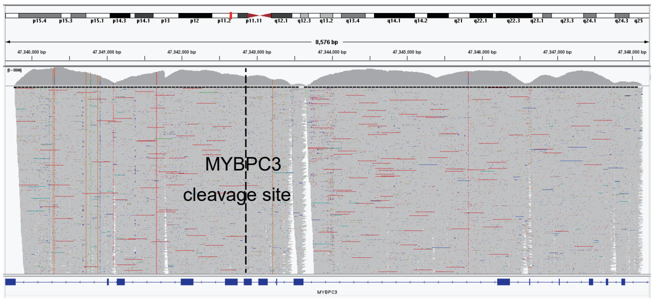

b

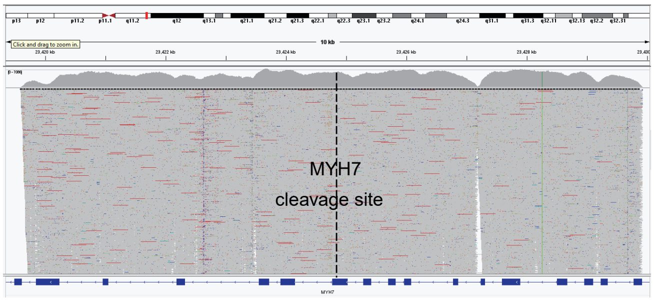

Supplementary Fig. 5: Illustration of genomic regions sequenced for assessment of LOH and detection of large deletions in established ESCs. **a.** Snapshot of the genomic region interrogated by sequencing of 8.3Kb PCR product covering *MYBPC3* cleavage site (Integrated Genome Viewer). **b.** Snapshot of the genomic region interrogated by sequencing of 10Kb PCR product covering *MYH7* cleavage site (Integrated Genome Viewer).

a

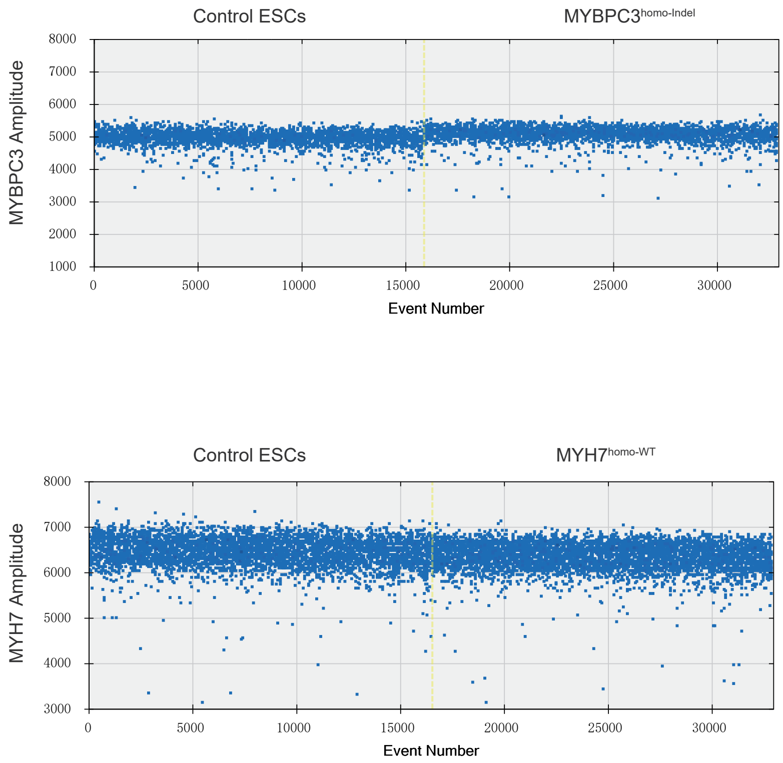

Supplementary Fig. 6: ddPCR assay for copy number variations in edited ESC clones. **a.** Copy number quantification by ddPCR assay for *MYBPC3* locus shows similar profiles for *MYBPC3*$^{homo\text{-}Indel}$ and control ESC lines. **b.** Copy number quantification by ddPCR assay for *MYH7* locus shows similar profiles for *MYH7*$^{homo\text{-}WT}$ and control ESC lines.

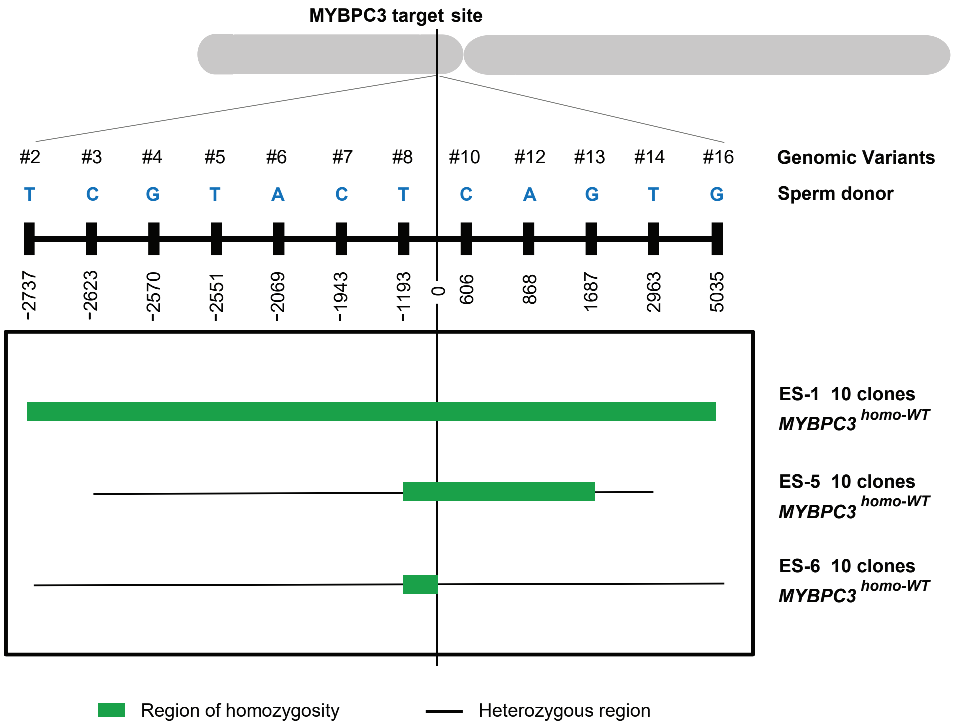

Supplementary Fig. 7: Genomic variants in *MYBPC3*$^{homo\text{-}WT}$ ESCs. Schematic map of 12 genomic variants located upstream and downstream at various distances from the target *MYBPC3* locus in *MYBPC3*$^{homo\text{-}WT}$ ESC clones from known sperm donor genotype but lack egg donor genotype. Green lines indicate that these loci are homozygous but LOH cannot be determined because genotypes of contributing oocyte donors were not available. Black lines indicate that these loci are heterozygous. Source Data are provided as Supplementary Table 1.

Supplementary Table 1. SNP genotypes at the *MYBPC3* locus in ESC lines lacking egg donor information.

Note: Green letters represent homozygous regions. Blue letters represent nucleotides of sperm donor.

Genome variant ID	Sperm donor	ES-1 10 clones	ES-5 10 clones	ES-6 10 clones
**#2**	T/T	**T/T**	n/a	T/T
**#3**	C/C	**C/C**	C/CACAG	C/CACAG
**#4**	G/G	**G/G**	G/G	G/G
**#5**	T/T	**T/T**	C/T	T/C
**#6**	A/A	**A/A**	G/A	G/A
**#7**	C/C	**C/C**	G/C	G/C
**#8**	T/T	**T/T**	**T/T**	**T/T**
**On target site**	WT/WT	**WT/WT**	**WT/WT**	**WT/WT**
**#10**	C/C	**C/C**	**C/C**	C/T
**#12**	A/A	**A/A**	**A/A**	A/G
**#13**	G/G	**G/G**	**G/G**	G/T
**#14**	T/T	**T/T**	T/C	T/C
**#16**	G/G	**G/G**	n/a	G/A
