# Supplementary material for: Limitations of gene editing assessments in human preimplantation embryos
Source: Nat Commun. 2023 Mar 7;14:1219. doi: 10.1038/s41467-023-36820-6 (PMC9992379; doi:10.1038/s41467-023-36820-6)
Supplement: Supplementary file 11 — Description of Additional Supplementary Files [file 41467_2023_36820_MOESM11_ESM.pdf]

Title: Supplementary Data 1.

Description: *MYBPC3* genotypes in individual blastomeres of injected embryos. Large deletions (del ≥100 bp) are highlighted in red; ins represents insertion; del represents deletion; sub represents substitution.

Title: Supplementary Data 2.

Description: *LDLRAP1* genotypes in blastomeres of edited and control embryos. Control blastomeres with LOH are highlighted in yellow.

Title: Supplementary Data 3.

Description: *MYH7* genotype in individual blastomeres of control non-injected embryos. Control blastomeres with LOH are highlighted in yellow.

Title: Supplementary Data 4.

Description: *MYH7* genotype in individual blastomeres of injected embryos. Large deletions (del ≥100 bp) are highlighted in red; ins represents insertion; del represents deletion; sub represents substitution.

Title: Supplementary Data 5.

Description: Sample-specific ADO rates in DNA after WGA.

Title: Supplementary Data 6.

Description: Locus-specific ADO rates in single cell DNA after WGA.

Title: Supplementary Data 7.

Description: Preimplantation development of CRISPR/Cas9 injected embryos.

Title: Supplementary Data 8.

Description: *MYBPC3* and *MYH7* genotypes in ESC subclones derived from edited embryos. Cell lines with large deletions are highlighted in yellow.

Title: Supplementary Data 9.

Description: Genome variants and LOH at *MYBPC3* locus in ESC clones. Green represents homozygous regions, red represents loss of paternal variants; yellow highlighted loci represent informative genomic variants; n/a - not applicable.
