# Supplementary material for: Limitations of gene editing assessments in human preimplantation embryos
Source: Nat Commun. 2023 Mar 7;14:1219. doi: 10.1038/s41467-023-36820-6 (PMC9992379; doi:10.1038/s41467-023-36820-6)
Supplement: Supplementary file 12 — Reporting Summary [file 41467_2023_36820_MOESM12_ESM.pdf]

nature portfolio

Corresponding author(s): Shoukhrat Mitalipov

Last updated by author(s): Feb 1, 2023

Reporting Summary

Nature Portfolio wishes to improve the reproducibility of the work that we publish. This form provides structure for consistency and transparency in reporting. For further information on Nature Portfolio policies, see our Editorial Policies and the Editorial Policy Checklist.

Statistics

For all statistical analyses, confirm that the following items are present in the figure legend, table legend, main text, or Methods section.

n/a	Confirmed	
☐	☒	The exact sample size (*n*) for each experimental group/condition, given as a discrete number and unit of measurement
☐	☒	A statement on whether measurements were taken from distinct samples or whether the same sample was measured repeatedly
☐	☒	The statistical test(s) used AND whether they are one- or two-sided<br>*Only common tests should be described solely by name; describe more complex techniques in the Methods section.*
☒	☐	A description of all covariates tested
☐	☒	A description of any assumptions or corrections, such as tests of normality and adjustment for multiple comparisons
☒	☐	A full description of the statistical parameters including central tendency (e.g. means) or other basic estimates (e.g. regression coefficient) AND variation (e.g. standard deviation) or associated estimates of uncertainty (e.g. confidence intervals)
☐	☒	For null hypothesis testing, the test statistic (e.g. *F*, *t*, *r*) with confidence intervals, effect sizes, degrees of freedom and *P* value noted<br>*Give P values as exact values whenever suitable.*
☒	☐	For Bayesian analysis, information on the choice of priors and Markov chain Monte Carlo settings
☒	☐	For hierarchical and complex designs, identification of the appropriate level for tests and full reporting of outcomes
☒	☐	Estimates of effect sizes (e.g. Cohen's *d*, Pearson's *r*), indicating how they were calculated

Our web collection on statistics for biologists contains articles on many of the points above.

Software and code

Policy information about availability of computer code

Data collection	ISIS (MetaSystem GmbH): FISH
Data analysis	SnapGene Viewer (v6.0.2): Sanger sequencing analysis; QuantaSoft Analysis Pro (Bio-Rad): ddPCR; Trim Galore (v0.6.3); BWA-MEM (v0.7.17); Picard tools (v2.18.2, v2.26.9); SAMtools (v1.10); FreeBayes (v1.3.1); Delly (v0.8.7); Lumpy (v0.3.1); R (v4.0.3); RStudio (v1.3.1093); IGV (2.11.3); Excel (v16.0.10392.20029); ISIS (MetaSystem GmbH): FISH

For manuscripts utilizing custom algorithms or software that are central to the research but not yet described in published literature, software must be made available to editors and reviewers. We strongly encourage code deposition in a community repository (e.g. GitHub). See the Nature Portfolio guidelines for submitting code & software for further information.

Data

Policy information about availability of data

All manuscripts must include a data availability statement. This statement should provide the following information, where applicable:

- Accession codes, unique identifiers, or web links for publicly available datasets
- A description of any restrictions on data availability
- For clinical datasets or third party data, please ensure that the statement adheres to our policy

Data supporting the findings of this study are included in this manuscript. The following publicly available datasets were used in this project: GRCh38 genome assembly. Amplicon sequencing data has been deposited in the Sequence Read Archive of the NCBI under the BioProject ID PRJNA909213 and are publicly available as of Jan 31st, 2023. Since whole-genome sequencing data can reveal the genetic identity of the study participants, OHSU IRB regulations, participant consent

forms, and Oregon laws prohibit us from sharing these data publicly.

Human research participants

Policy information about studies involving human research participants and Sex and Gender in Research.

Reporting on sex and gender	Male and female gamete, blood and skin fibroblast donors were recruited for this study. The sex of the embryos was not determined or taken into account as part of the study design.
Population characteristics	Two adult sperm donors 28-51 years of age with certain genetic mutation in MYH7 or LDLRAP1 genes were identified and enrolled in this study.<br><br>Thirteen healthy oocyte donors of 21-35 years of age that met inclusion criteria for egg donation were enrolled in this study:<br>• age 21-30 years old;<br>• Regular menstrual cycles every 21-35 days;<br>• Not pregnant;<br>• Be willing and able to sign informed consent and travel to one of the participating IVF clinics for trail related visits and procedures;<br>• Willing to abstain from intercourse or use barrier method contraception during the stimulation cycle of their study participation.<br><br>Exclusion:<br>• Being diagnosed with polycystic ovarian syndrome (PCOS);<br>• BMI (body mass index) over 28;<br>• Any health conditions that might increase risk of participation, such as endocrine disorders, hypertension, diabetes, heart disease, autoimmune disorders, known allergy to anesthesia (lidocaine);<br>• Current medical history of an active psychiatric disorder;<br>• Significant bleeding disorder that might make a biopsy procedure unsafe;<br>• Any clinical or laboratory findings by the PIs that leads them to believe that candidate is not suitable for egg donation (e.g. anti-mullerian levels too low.<br><br>Five women with infertility undergoing IVF donated discarded immature oocytes for this study .<br><br>A family consisting of an adult female proband and both here parents were recruited as skin fibroblast donors for SCNT and allele dropout experiments.
Recruitment	Recruitment was organized via print and web-based advertising. All research gamete donors met required inclusion criteria. There is potential bias for gamete donors without access to the Internet. Written informed consent was obtained from all subjects prior to enrollment in the study. Subjects were informed of risks to participation including risks associated with clinical procedures and loss of confidentiality. All consent forms included a lay language summary of germline gene correction and somatic cell nuclear transfer, the ethical sensitivities surrounding germline gene modifications, and discussed the potential for incidental findings (genetic information potentially important to their future healthcare). Perspective participants were provided a copy of the consent form to review in advance of an in-person consent signing where the form is presented and discussed in further detail.
Ethics oversight	The study was approved by the OHSU Institutional Review Board (IRB) and included independent review by the OHSU Innovative Research Advisory Panel (IRAP) and OHSU Scientific Review Committee (SRC). The approved studies are subjected to bi-annual external regulatory monitoring and Data Safety Monitoring Committee (DSMC) reviews and annual IRB continuing review. Quantity of oocytes used in research studies are strictly regulated by DSMC/IRB and must be justified; all studies are required to use the minimal number of oocytes necessary to answer research questions.

Note that full information on the approval of the study protocol must also be provided in the manuscript.

Field-specific reporting

Please select the one below that is the best fit for your research. If you are not sure, read the appropriate sections before making your selection.

[x] Life sciences [ ] Behavioural & social sciences [ ] Ecological, evolutionary & environmental sciences

For a reference copy of the document with all sections, see nature.com/documents/nr-reporting-summary-flat.pdf

Life sciences study design

All studies must disclose on these points even when the disclosure is negative.

Sample size	No sample size calculations were performed. Sample size was chosen based on previously published work (Ma et al., 2017) and was reviewed by OHSU Data Safety Monitoring Committee and approved by OHSU Institutional Review Board. A total of 371 mature oocytes was used for this study
Data exclusions	No data were excluded from the analysis.
Replication	Results of on-target edits have been confirmed in multiple embryos (biological samples) derived from several different gamete donors (independent experiments).
Randomization	Zygotes and MII oocytes were randomly assigned into CRISPR/Cas9 injected and non-injected control groups. Individual sperm for ICSI was randomly picked up and injected into the oocytes.
Blinding	During all analyses , the personnel involved was blinded regarding the sample origin.

Reporting for specific materials, systems and methods

We require information from authors about some types of materials, experimental systems and methods used in many studies. Here, indicate whether each material, system or method listed is relevant to your study. If you are not sure if a list item applies to your research, read the appropriate section before selecting a response.

Materials & experimental systems

n/a	Involved in the study	
☒	☐	Antibodies
☐	☒	Eukaryotic cell lines
☒	☐	Palaeontology and archaeology
☒	☐	Animals and other organisms
☒	☐	Clinical data
☒	☐	Dual use research of concern

Methods

n/a	Involved in the study	
☒	☐	ChIP-seq
☒	☐	Flow cytometry
☒	☐	MRI-based neuroimaging

Eukaryotic cell lines

Policy information about cell lines and Sex and Gender in Research

Cell line source(s)	A total of 16 ESC lines was established for this study from edited and control human embryos.<br><br>Dermal fibroblast cell lines were generated from an adult female and two her parents for SCNT and allele dropout experiments.
Authentication	Sanger sequencing, G-banding karyotyping, Fish and Short tandem repeats.
Mycoplasma contamination	All cell lines were tested for Mycoplasma and appeared negative.
Commonly misidentified lines (See ICLAC register)	No commonly misidentified cell lines were used.
